# Supplementary material for: Research on the evaluation and impact trends of China’s skill talent ecosystem in the digital era – An analysis based on neural network models and PVAR models
Source: PLoS One. 2024 Jun 28;19(6):e0302909. doi: 10.1371/journal.pone.0302909 (PMC11213331; doi:10.1371/journal.pone.0302909)
Supplement: Attachment — Submitted filename: Response to Reviewers.doc [file pone.0302909.s002.doc]

**Response to Reviewers：

Q1: Mention the significance of coupling coordination model.**
Supplemented the importance and rationale for using the coupling coordination model.

**Q2: Figures 3 and 4 are incomplete. Mark X and Y axis properly.**

Figures 3 and 4 have been corrected, and the X and Y axes have been added.

**Q3: Add few recent references related to Neural Network and PVAR models.**

Relevant literature on the PVAR model and neural network model has been added.

**Q4: Compare with other works**

In the conclusion analysis section, compare and analyze the research conclusions of other scholars.

Summary:

Add more relevant literature.

Update the reference list.

Compare and analyze results with those of related papers.

Make language adjustments and improvements.

Correct any imperfections in figures and tables.

**Thank you for your expert advice!**
